# Water temperature shapes amino acid metabolism to modulate growth and development in crustacean larvae

**DOI:** 10.3389/fmicb.2026.1840429

**Published:** 2026-05-20

**Authors:** Siqi Wang, Peng Huang, Jiancao Gao, Jinliang Du, Yu Yao, Wenyong Chen, Gangchun Xu

**Affiliations:** 1 Wuxi Fisheries College, Nanjing Agricultural University, Wuxi, China; 2 Freshwater Fisheries Research Center, Chinese Academy of Fishery Sciences, Wuxi, China; 3 Sheyang Chengxin Yangtze River Crab Ecological Breeding Farm, Yancheng, China

**Keywords:** amino acid metabolism, *Eriocheir sinensis* larvae, growth and development, holobiont, water temperature

## Abstract

Water temperature is a key environmental factor influencing the growth and development of crustaceans, yet the mechanisms underlying larval responses to temperature remain poorly understood. This study investigated the effects of temperature variation on holobiont interactions in larvae of the *Eriocheir sinensis*. Larvae were exposed to a normal temperature regime (14 °C–23 °C, CON) or a reduced temperature regime (14 °C–19 °C LT) for 14 days. Lower temperature significantly inhibited larval growth and development, as evidenced by reduced overall length, head length, dorsal spine length, and frontal spine length (*P* < 0.05). Biochemical analyses revealed increased alkaline phosphatase (AKP) activity but decreased levels of alanine aminotransferase (ALT), aspartate aminotransferase (AST), total protein (TP), glucose (GLU), and triglycerides (TG), suggesting suppressed metabolic activity and energy utilization under low temperature. Metabolomic analysis showed pronounced metabolic reprogramming, with 557 metabolites enriched in the CON group and 802 in the LT group, mainly associated with lysine biosynthesis and degradation, arginine biosynthesis, and phenylalanine, tyrosine, and tryptophan metabolism. Temperature shifts also reshaped the larval microbiota. The relative abundances of *Actinomycetota*, *Bacteroidota*, and *Thermodesulfobacteriota* and several genera decreased under LT, whereas *Leucothrix*, *Marivita*, *Yoonia*, and *Pseudoalteromonas* increased (*P* < 0.05). Microbial functional potentials related to amino acid metabolism were significantly suppressed. Overall, these findings suggest that reduced temperature alters amino acid metabolic capacity and host–microbiota interactions, ultimately constraining the growth and development of *E. sinensis* larvae.

## Introduction

Water temperature is a critical environmental factor governing crustacean growth, development, metabolic programming, and survival (Pilakouta et al., 2023; Wolfe et al., 2024). Even subtle variations in ambient temperature can profoundly influence host physiological processes, particularly during early developmental stages, when organisms display elevated metabolic plasticity and heightened responsiveness to environmental stressors (Chen et al., 2025; Scott and Johnston, 2012; Stillson et al., 2025). In crustaceans, the larval stage is marked by rapid tissue differentiation and elevated nutritional requirements, rendering the larvae especially sensitive to fluctuations in water temperature (Lemos et al., 2003). Previous studies have shown that crustaceans largely depend on ambient temperature to sustain physiological homeostasis (Clark et al., 2017; Crabbe, 2009). Temperature modulates host energy allocation (Griffen, 2017), metabolic capacity (van der Walt et al., 2025), immune responses (Liu et al., 2018), and somatic development (Paganini et al., 2014). These findings indicate that temperature plays a pivotal role in shaping early-life processes in crustaceans. Therefore, elucidating how aquatic crustaceans respond to temperature fluctuations is critical for advancing our understanding of environment–host interactions.

Under suboptimal thermal conditions, *Eriocheir sinensis* larvae exhibit delayed molting, suppressed feeding behavior, and reduced growth rates (Deng et al., 2024; Raby et al., 2025; Yin et al., 2025). Previous research has largely centered on the impacts of temperature on adult crustaceans, encompassing growth performance (Aguilar-Alberola and Mesquita-Joanes, 2014), immune function (Garcia-Rueda et al., 2023), and molting regulation (Andersen et al., 2022; Sun, 2025). However, the underlying metabolic mechanisms through which temperature governs larval growth remain largely unexplored, particularly regarding the regulatory roles of holobiont (A dynamic whole composed of host cells and microbial cells) and metabolism in early-stage larvae. Accumulating evidence demonstrates that crustacean holobiont are pivotal for nutrient assimilation and energy metabolism (Brante et al., 2025; Ishaq et al., 2023; Lu et al., 2024; Yang et al., 2024). Therefore, elucidating the water temperature–metabolic programming–microbiota axis offers new insights and a conceptual framework for understanding the mechanisms by which temperature influences larval metabolic programming in crustaceans.

Amino acid metabolism constitutes a fundamental cornerstone in larval growth, not only supplying substrates for protein synthesis but also contributing to energy production and regulating signaling pathways associated with development and stress adaptation (Jiang et al., 2024; Zhou J. et al., 2022). In crustaceans, amino acids act not only as structural constituents but also as key modulators of metabolic homeostasis, especially under conditions of environmental stress (Zou et al., 2025). Recent studies have demonstrated that temperature fluctuations can reprogram multiple metabolic pathways, including amino acid biosynthesis and catabolism, consequently influencing growth efficiency (Liu et al., 2023). Nevertheless, systematic studies examining how temperature modulates amino acid metabolism in *E. sinensis* larvae, and how such modulation impacts growth, remain scarce. Based on these considerations, we hypothesized that lowered temperatures constrain amino acid metabolism, impair protein synthesis, and thereby reduce the growth rate of larvae. To test this hypothesis, we integrated 16S rRNA sequencing, untargeted metabolomics, and metagenomic analyses to explore how temperature affects larval growth and physiological adaptation in *E. sinensis*. By elucidating the intrinsic connections between temperature-dependent metabolic regulation and larval growth, this study provides novel insights into the metabolic sensitivity of crustacean larvae to temperature fluctuations and furnishes scientific guidance for optimizing thermal management strategies in crustacean aquaculture.

## Materials and methods

### Animal experiments and sampling

The experiment was conducted at Sheyang Chengxin Yangtze River Crab Ecological Breeding Farm (Yancheng, China). Broodstock crabs were sourced from the Germplasm Resource Conservation Base at the Freshwater Fisheries Research Center, Chinese Academy of Fishery Sciences. Pre-hatch, ovigerous female *E. sinensis* were selected and placed in 100-L white polyethylene hatching tanks to allow for natural spawning. Newly hatched Zoea I-stage larvae were collected to serve as experimental subjects. The experiment consisted of a normal temperature (CON) and a low-temperature treatment group (LT). Each group comprised three replicates, yielding a total of six experimental units (rearing tanks). Larvae were randomly distributed across experimental units, with initial densities standardized. Both groups were initially maintained at 14 °C and subjected to gradual temperature elevation over a 14-day period. At the end of the 14-day period, temperatures were increased to 23 °C for the CON group (temperature was increased at an average rate of 0.65 °C/d) and 19 °C for the LT group (temperature was increased at an average rate of 0.35 °C/d). Larvae from each experimental unit were then collected, rapidly frozen in liquid nitrogen, and stored for subsequent analyses.

### Larval growth parameter assessment

The influence of temperature on larval developmental progression was examined under an optical microscope, with measurements taken for total length, head length, dorsal spine length, and frontal spine length.

### Larval biochemical parameter measurement

Each group consisted of nine independent biological replicates. Samples from different temperature treatments were weighed, and approximately 100 mg of tissue from each replicate was mixed with nine volumes of physiological saline (0.86%) to prepare a 10% tissue homogenate. The mixture was then homogenized in a grinding system at 4 °C. After homogenization, the mixture was centrifuged at 5,000 rpm for 10 min, and the supernatant was collected. The activities and concentrations of alkaline phosphatase (AKP), alanine aminotransferase (ALT), acid phosphatase (ACP), aspartate aminotransferase (AST), total protein (TP), triglycerides (TG), glucose (GLU), low-density lipoprotein cholesterol (LDL-C), and high-density lipoprotein cholesterol (HDL-C) were quantified in strict accordance with the manufacturer’s instructions (Nanjing Jiancheng Bioengineering Institute, Nanjing, China).

### Untargeted metabolomics analysis

Each group comprised nine independent biological replicates. Equal-mass tissue samples were placed in 2 mL centrifuge tubes, to which grinding beads and 400 μL of extraction solvent (ethanol:water, 4:1, v/v) were added. Samples were frozen and homogenized for 6 min, followed by ultrasonication at 5 °C for 30 min to extract metabolites. Following a 30-min equilibration, samples were centrifuged at 13,000 × *g* for 15 min, and the resulting supernatant was collected for subsequent analysis. Metabolite profiling was conducted using a UHPLC-Q Exactive HF-X system (Thermo Fisher Scientific). Chromatographic separation was performed on an HSS T3 column (100 × 2.1 mm, 1.8 μm) with a 3 μL injection volume, a flow rate of 0.40 mL/min, and a column temperature of 40 °C. Mobile phase A was composed of water:acetonitrile (95:5, v/v) containing 0.1% formic acid, whereas mobile phase B comprised acetonitrile:isopropanol:water (47.5:47.5:5, v/v) with 0.1% formic acid. All solvents were filtered through 0.22 μm membranes prior to use. Raw LC-MS data were preprocessed using the Progenesis QI platform (Waters), including baseline correction, chromatographic peak alignment, and feature extraction, resulting in a three-dimensional matrix of retention time, mass-to-charge ratio, and peak intensity. Metabolite identification was performed by matching against the HMDB and Metlin databases. Preprocessed data were uploaded to the Majorbio cloud platfor^1^ for further analysis.

### 16 S rRNA gene sequencing

Bacterial genomic DNA was extracted in accordance with the manufacturer’s instructions using the FastDNA^®^ Spin Kit for Soil (MP Biomedicals, Southern California, United States). DNA concentrations were measured using a NanoDrop 2000 spectrophotometer (Thermo Fisher Scientific, United States), and DNA integrity was evaluated by agarose gel electrophoresis. Following DNA quality assessment, universal primers 338F (5′-ACT CCT ACG GG AGG CAG CAG AG-3′) and 806R (5′-GGA CTA CHV GGG TWT CTA AT-3′) were used for PCR amplification of the *E. sinensis* holobiont 16S rRNA genes with TransGen AP221-02 and TransStart FastPfu DNA polymerase. PCR products were assessed on a 2% agarose gel, and the target amplicons were purified using the AxyPrep DNA Gel Extraction Kit (Axygen Biosciences, United States). Purified DNA concentrations were measured using the Qubit dsDNA Assay Kit (Thermo Fisher Scientific, United States). Sequencing was conducted on an Illumina NovaSeq 6000 platform, generating paired-end reads. Raw paired-end reads were merged, quality-filtered, and denoised into amplicon sequence variants (ASVs), followed by taxonomic assignment. Microbial α-diversity indices, including Chao, Ace, Shannon, and Simpson indices, were calculated to assess holobiont diversity. Linear discriminant analysis effect size (LEfSe) was employed to identify differences in the relative abundance of bacterial taxa. Multivariate analyses and statistical significance testing were performed to compare microbial community composition and phylogenetic relationships among samples. And adopt the method of Xun et al. (2021) to calculate the Average Variation Degree (AVD) of microorganisms.

### Metagenomic analysis

Genomic DNA was extracted from samples using the E.Z.N.A. Soil DNA Kit (Omega Bio-Tek, United States) following the manufacturer’s instructions. DNA quality and concentration were assessed using a NanoDrop spectrophotometer (Thermo Fisher Scientific, Wilmington, DE, United States), and DNA integrity was evaluated by 1% agarose gel electrophoresis. DNA was sheared using a Covaris M220 system (China Gene Company), and paired-end sequencing libraries were constructed with the NEXTFLEX Rapid DNA Sequencing Kit (Bioo Scientific, Austin, TX, United States). Reads were quality-filtered to remove sequences in which >50% of bases had a quality score <20 or that contained >10% ambiguous nucleotides, resulting in high-quality, clean reads. Host-derived reads were removed using Bowtie2 to minimize contamination. High-quality reads were assembled de novo using MEGAHIT (Li et al., 2015), with k-mer values set between 21 and 99. Unmapped reads from each sample were merged and reassembled to create co-assemblies. Assembly quality was assessed using BWA (Li and Durbin, 2009). Open reading frames (ORFs) exceeding 500 bp were predicted from assembled contigs using MetaGeneMark (Zhu et al., 2010). Predicted ORFs were clustered using CD-HIT (Li and Godzik, 2006) with a 95% sequence identity threshold and 90% read coverage to construct a non-redundant gene catalog. Sequencing reads were mapped to the non-redundant gene catalog, and gene abundances were quantified using SOAPaligner (Li et al., 2008) at 95% sequence identity. The non-redundant genes were functionally and taxonomically annotated by comparison against the NCBI NR database. Predicted non-redundant genes were aligned to the CAZy database^2^ using Diamond (v0.9.14.115) (Buchfink et al., 2015) with the blastx algorithm and an E-value threshold of 1 × e^–5^. Gene functional annotations and metabolic pathway assignments were performed using eggnog-mapper (v2.1.3) to assign KEGG orthology terms, providing insights into gene functions and metabolic pathways.

### Statistical analysis

All statistical analyses were performed using SPSS 21.0 (IBM Corp., Armonk, NY, United States), with a *P*-value ≤ 0.05 considered statistically significant. Differences between groups were assessed using an independent-samples *T*-test for normally distributed data or the Kruskal-Wallis test otherwise.

## Result

### Growth and development of *E. sinensis* larvae are restricted under low-temperature conditions

**FIGURE 1:**
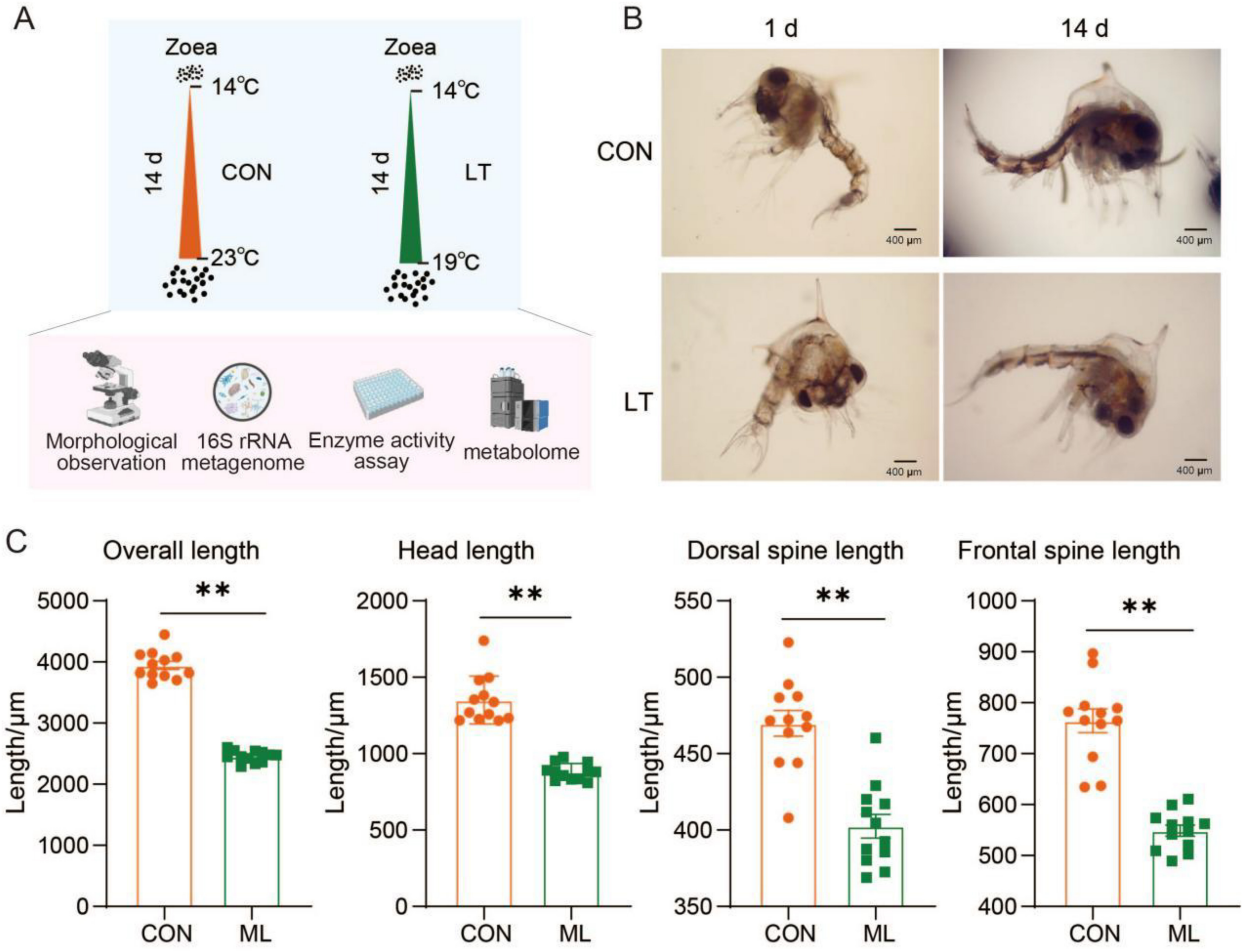
Effects of temperature on larval development in *Eriocheir sinensi*s. **(A)** Schematic overview of the experimental design. **(B)** Morphological characteristics of larvae exposed to different temperature conditions. **(C)** Comparative analysis of larval growth parameters. “**” indicates *P* < 0.01. CON, normal temperature group; LT, low-temperature group.

In this study, we established a temperature-driven developmental model for *E. sinensis* larvae using a gradual warming regime (Figure 1A). We found that reduced temperature constrained larval developmental progression, with a higher proportion of larvae reaching the zoea III stage under low temperature, whereas larvae reared at normal temperature progressed to the zoea IV stage (*P* < 0.05, Figure 1B). Furthermore, larvae exposed to lower temperature exhibited significantly reduced overall length, head length, dorsal spine length, and frontal spine length compared with those maintained at normal temperature (*P* < 0.05, Figure 1C). Collectively, these results indicate that reduced temperature restricts the growth and development of *E. sinensis* larvae.

### Temperature alters host enzymatic activities in *E. sinensis* larvae

**FIGURE 2:**
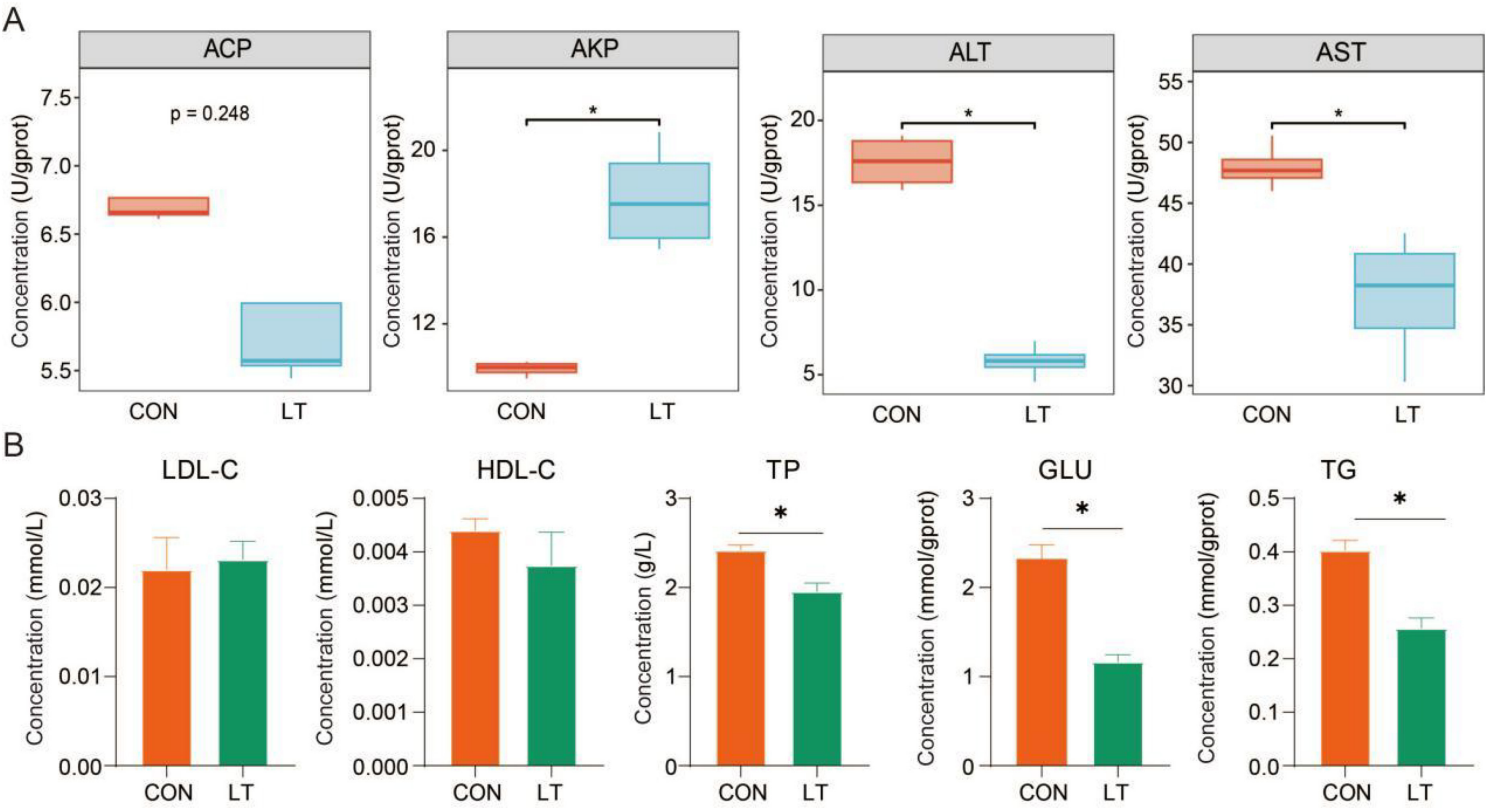
Effects of temperature variation on host enzyme activities in *Eriocheir sinensis* larvae. **(A)** Enzymatic indicators related to tissue metabolic function. **(B)** Activities of energy-related metabolic enzymes. “*” indicates *P* < 0.05. CON, normal temperature group; LT, low-temperature group.

Host enzymatic activities play a critical role in maintaining metabolic homeostasis. We found that exposure to lower temperature resulted in elevated AKP levels and reduced ALT and AST levels in *E. sinensis* larvae (*P* < 0.05; Figure 2A). Consistently, the concentrations of TP, GLU, and TG were significantly decreased in larvae reared under lower temperature conditions (*P* < 0.05; Figure 2B). These results indicate that low temperature constrains metabolic activity and energy utilization in *E. sinensis* larvae.

### Changes in temperature drive metabolic flux reprogramming in *E. sinensis* larvae

**FIGURE 3:**
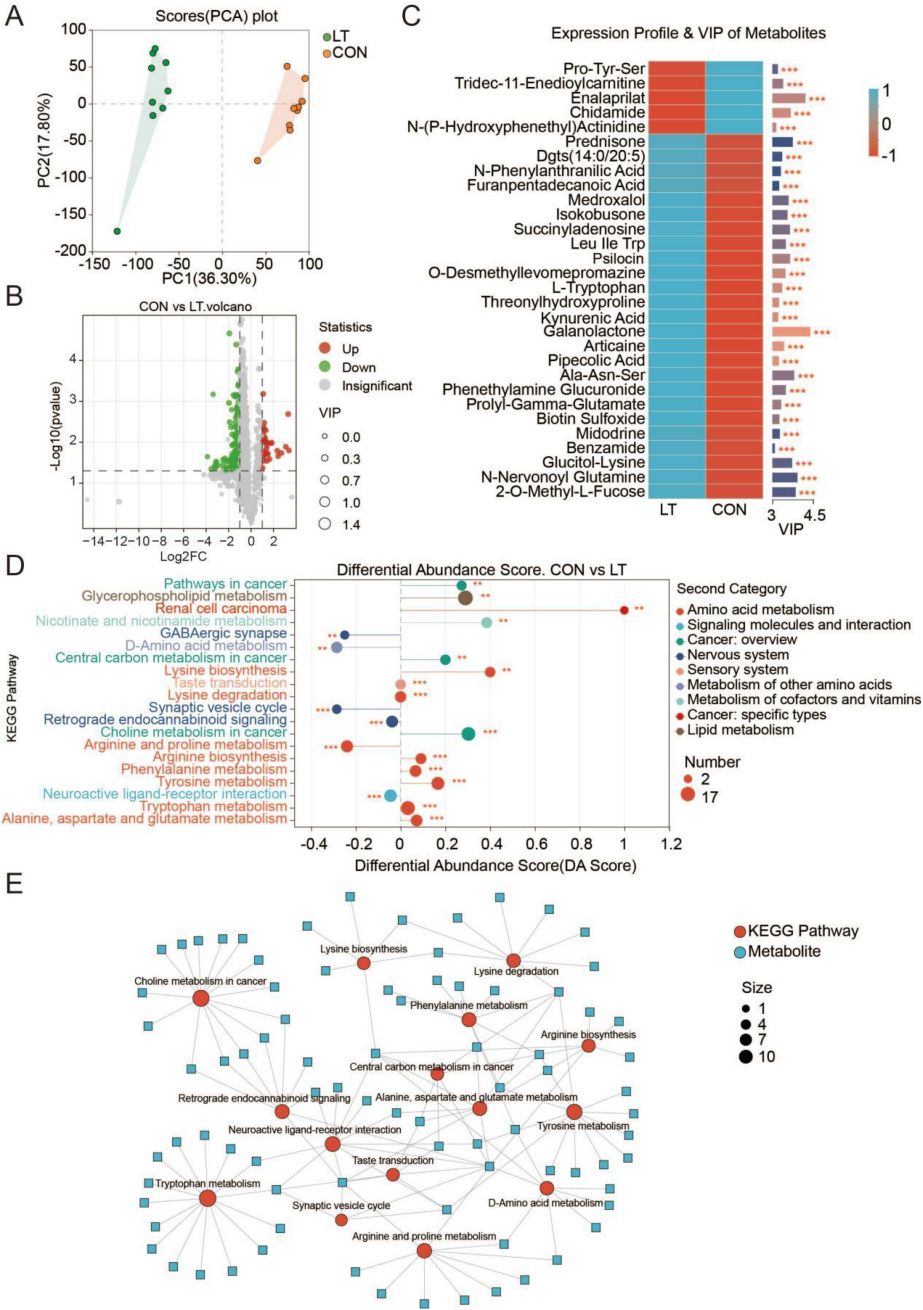
Metabolic pathway remodeling in *Eriocheir sinensis* larvae induced by temperature variation. **(A)** Scores plot of principal component analysis (PCA) based on metabolite profiles. **(B)** Volcano plot of differential metabolites, with significance thresholds set at *P* < 0.05 and | Log_2_FC | > 1.5. **(C)** Heatmap of the top 50 significantly altered metabolites. **(D)** Metabolic pathway enrichment analysis based on the identified differential metabolites. **(E)** Reconstructed metabolic network highlighting pathways remodeled by temperature. CON, normal temperature group; LT, low-temperature group. “*” indicates *P* < 0.05; “**” indicates *P* < 0.01.

Untargeted metabolomics was employed to investigate temperature-driven alterations in the metabolic profiles of *E. sinensis* larvae. Principal component analysis (PCA) revealed a clear separation in metabolite composition between larvae reared under different temperature conditions (Figure 3A). Specifically, 557 metabolites were enriched in the normal-temperature group, whereas 802 metabolites were enriched in the lower-temperature group (Figure 3B). Furthermore, differential metabolite analysis was performed using a threshold of VIP > 3. Pro-Tyr-Ser, Tridec-11-Enedioylcarnitine, Enalaprilat, Chidamide, and N-(p-hydroxyphenethyl)actinidine were enriched in the lower-temperature group, whereas Prednisone Galanolactone, N-nervonoyl glutamine, and 2-O-methyl-L-fucose were significantly enriched in the normal-temperature group (Figure 3C). Differential metabolite classification revealed that the majority were associated with amino acid metabolism. Under normal temperature, enriched metabolites were primarily linked to lysine biosynthesis, lysine degradation, arginine biosynthesis, phenylalanine metabolism, tyrosine metabolism, tryptophan metabolism, and alanine, aspartate, and glutamate metabolism, whereas those enriched under lower temperature were predominantly associated with arginine and proline metabolism (Figure 3D). Correlation network analysis yielded similar findings, indicating that temperature variations primarily drive lysine biosynthesis, lysine degradation, phenylalanine metabolism, arginine biosynthesis, tyrosine metabolism, tryptophan metabolism, D-amino acid metabolism, arginine and proline metabolism, and alanine, aspartate, and glutamate metabolism (Figure 3E). Collectively, these results indicate that temperature variation drives metabolic remodeling in *E. sinensis* larvae, with these changes predominantly involving amino acid metabolism.

### Temperature reshapes the holobiont ecology of *E. sinensis* larvae

**FIGURE 4:**
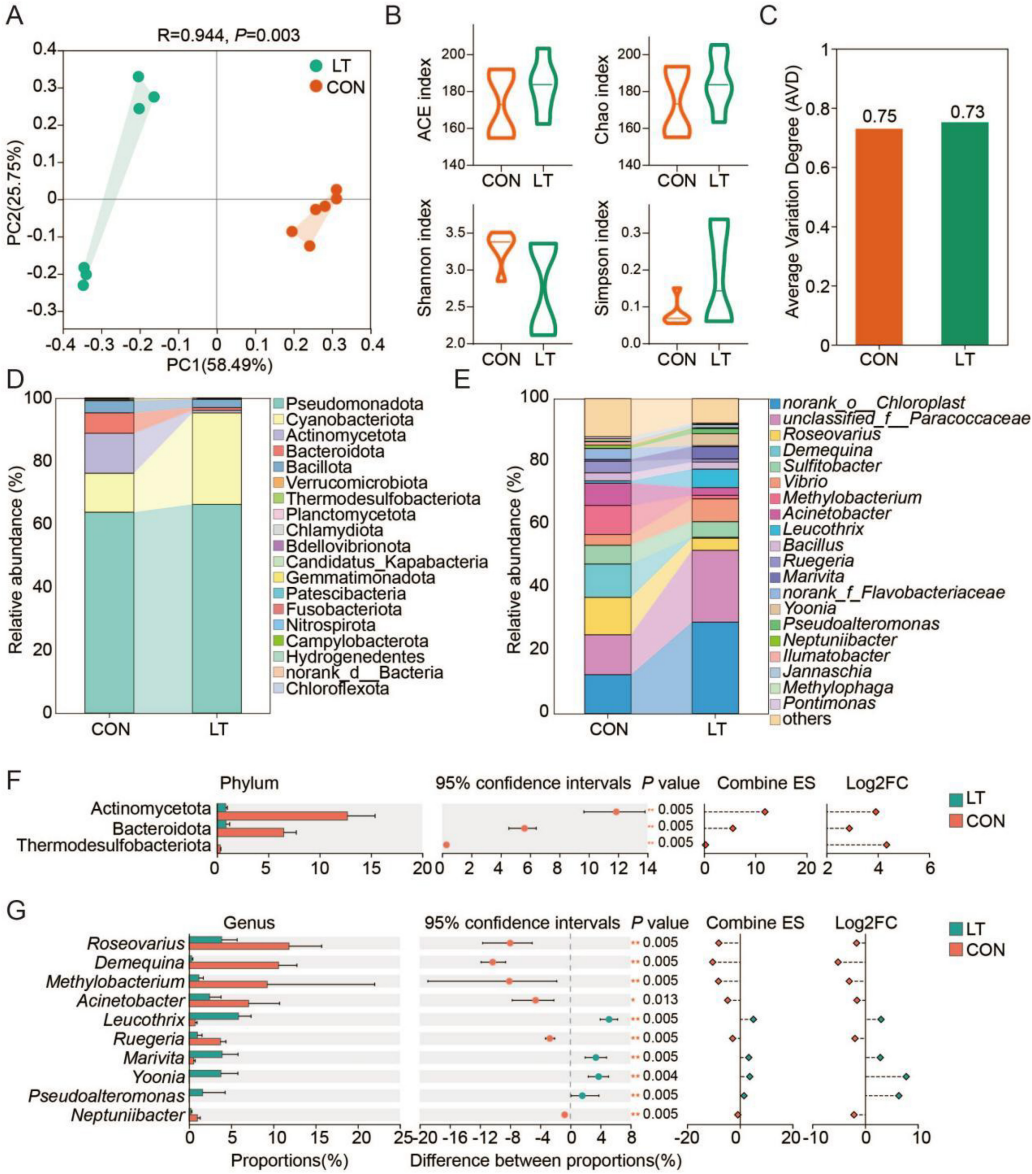
Temperature variation drives the reshaping of the holobiont in *Eriocheir sinensis* larvae. **(A)** Principal coordinate analysis (PCoA) of bacterial community composition based on amplicon sequence variants (ASVs). **(B)** Comparison of α-diversity indices of the bacterial communities. **(C)** Effect of temperature on bacterial community stability. **(D)** Microbial composition at the phylum level. **(E)** Microbial composition at the genus level. **(F)** Differential analysis of microbial composition at the phylum level. **(G)** Differential analysis of microbial composition at the genus level. “*” indicates *P* < 0.05; “**” indicates *P* < 0.01. CON, normal temperature group; LT, low-temperature group.

Holobiont play a pivotal role in host metabolic programming. PCA revealed that the holobiont composition of *E. sinensis* larvae was significantly influenced by temperature (Figure 4A). However, bacterial α-diversity, as assessed by ACE, Shannon, Chao, and Simpson indices, did not differ significantly between temperature groups (*P* > 0.05; Figure 4B). Moreover, the Average Variation Degree (AVD) index was elevated in larvae exposed to lower temperature (Figure 4C). At the phylum level, the holobiont was predominantly composed of *Pseudomonadota*, *Cyanobacteriota*, *Actinomycetota*, *Bacteroidota*, *Bacillota*, *Verrucomicrobiota*, *Thermodesulfobacteriota*, *Planctomycetota*, *Chlamydiota*, *Bdellovibrionota*, *Candidatus_Kapabacteria*, *Gemmatimonadota*, *Patescibacteria*, *Fusobacteriota*, *Nitrospirota*, *Campylobacterota*, *Hydrogenedentes*, *norank_d__Bacteria*, and *Chloroflexota* (Figure 4D). At the genus level, the community was dominated by *norank_o__Chloroplast*, unclassified_f__*Paracoccaceae*, *Roseovarius*, *Demequina*, *Sulfitobacter*, *Vibrio*, *Methylobacterium*, *Acinetobacter*, *Leucothrix*, *Bacillus*, *Ruegeria*, *Marivita*, norank_f__*Flavobacteriaceae*, *Yoonia*, *Pseudoalteromonas*, *Neptunibacter*, *Ilumatobacter*, *Jannaschia*, *Methylophaga*, and *Pontimonas* (Figure 4E). Further analysis showed that, at the phylum level, exposure to lower temperature significantly reduced the relative abundance of *Actinomycetota*, *Bacteroidota*, and *Thermodesulfobacteriota* (Figure 4F). At the genus level, *Roseovarius*, *Demequina*, *Methylobacterium*, *Acinetobacter*, *Ruegeria*, and *Neptunibacter* were enriched in larvae reared at normal temperature, whereas *Leucothrix*, *Marivita*, *Yoonia*, and *Pseudoalteromonas* were enriched under lower temperature conditions (Figure 4G).

**FIGURE 5:**
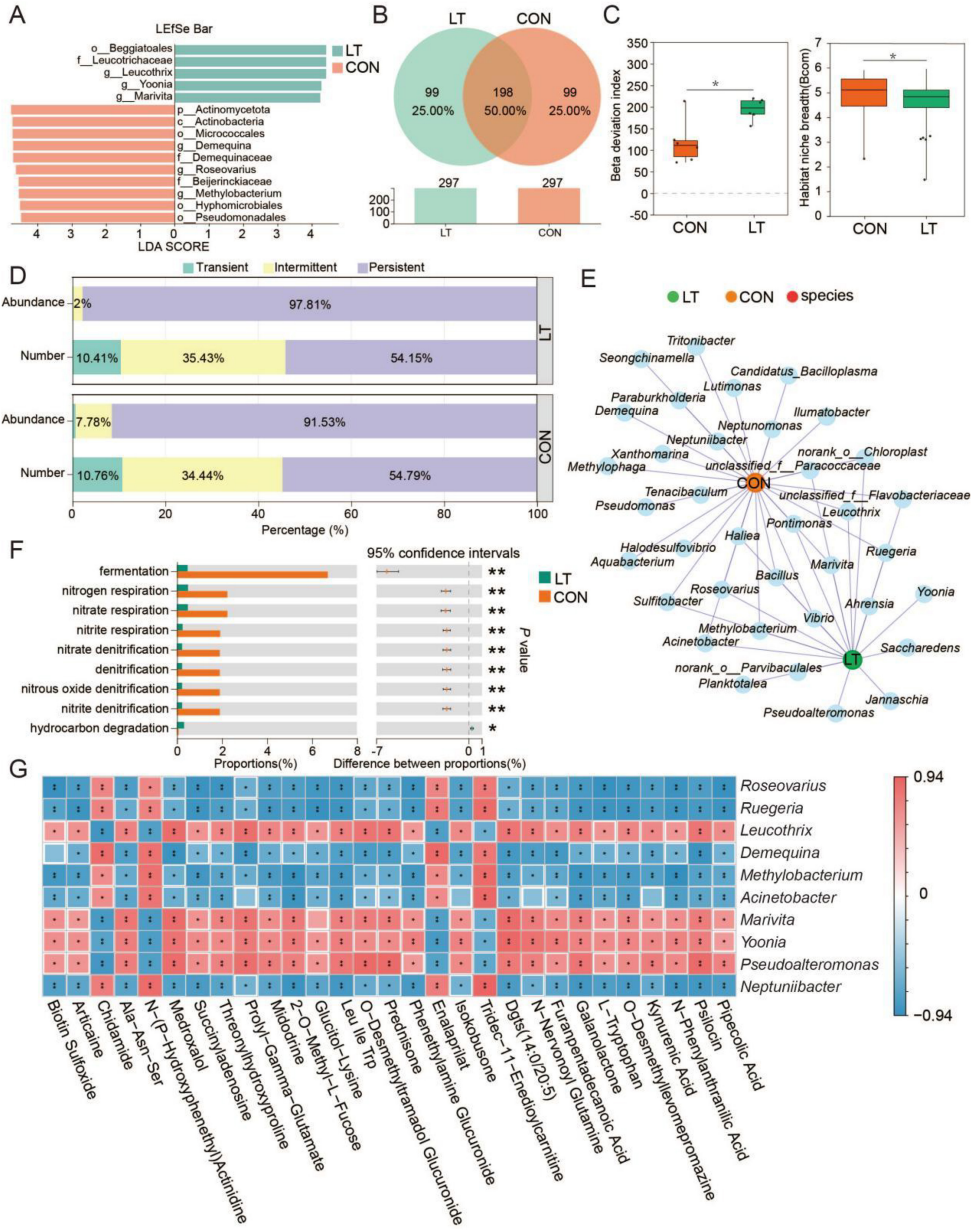
Temperature variation alters holobiont function and co-occurrence networks in *Eriocheir sinensis* larvae. **(A)** Differentially abundant taxa identified by LEfSe analysis at the species level. **(B)** Comparison of unique ASVs between temperature groups. **(C)** Comparison of microbial ecological niches characteristics. **(D)** Temperature sensitivity of core microbial taxa. **(E)** Co-occurrence networks of microbial communities under different temperature conditions. **(F)** Comparative analysis of predicted microbial functions. **(G)** Correlation heatmap of microorganisms and metabolites. “*” indicates *P* < 0.05; “**” indicates *P* < 0.01. CON, normal temperature group; LT, low-temperature group.

Linear discriminant analysis (LDA) score analysis indicated that o_*Beggiatoales*, f_*Leucotrichaceae*, g_*Leucothrix*, g_*Yoonia*, and g_*Marivita* were enriched under lower temperature, whereas p__*Actinomycetota*, c__*Actinobacteria*, o__*Micrococcales*, g__*Demequina*, f__*Demequinaceae*, g__*Roseovarius*, f__*Beijerinckiaceae*, g__*Methylobacterium*, o__*Hyphomicrobiales*, and o__*Pseudomonadales* were enriched at normal temperature (Figure 5A). Concurrently, 99 ASVs were unique to the lower-temperature group, while another 99 ASVs were specific to the normal-temperature group (Figure 5B). In addition, the Beta deviation index was significantly elevated under lower temperature, while the Habitat niche breadth index was significantly reduced (*P* < 0.05, Figure 5C). Furthermore, lower temperature conditions were associated with an increased proportion of persistent microbes and a decreased proportion of intermittent microbes in the larval holobiont (Figure 5D). More specifically, *Sulfitobacter*, *Roseovarius*, *Bacillus*, *Marivita*, *Leucothrix*, *Acinetobacter*, *Methylobacterium*, and *Vibrio* were identified as key taxa responding to temperature variations (Figure 5E). Alterations in these microbial taxa corresponded with functional shifts in the community, where fermentation, nitrogen respiration, nitrate respiration, nitrite respiration, nitrate denitrification, denitrification, nitrous oxide denitrification, and nitrite denitrification were significantly inhibited, whereas hydrocarbon degradation was enhanced under lower temperature (Figure 5F). Moreover, the correlation results also revealed a significant correlation between changes in microorganisms and changes in metabolites (Figure 5G). Collectively, these results indicate that reduced temperature attenuates nitrogen-associated microbial functions.

**FIGURE 6:**
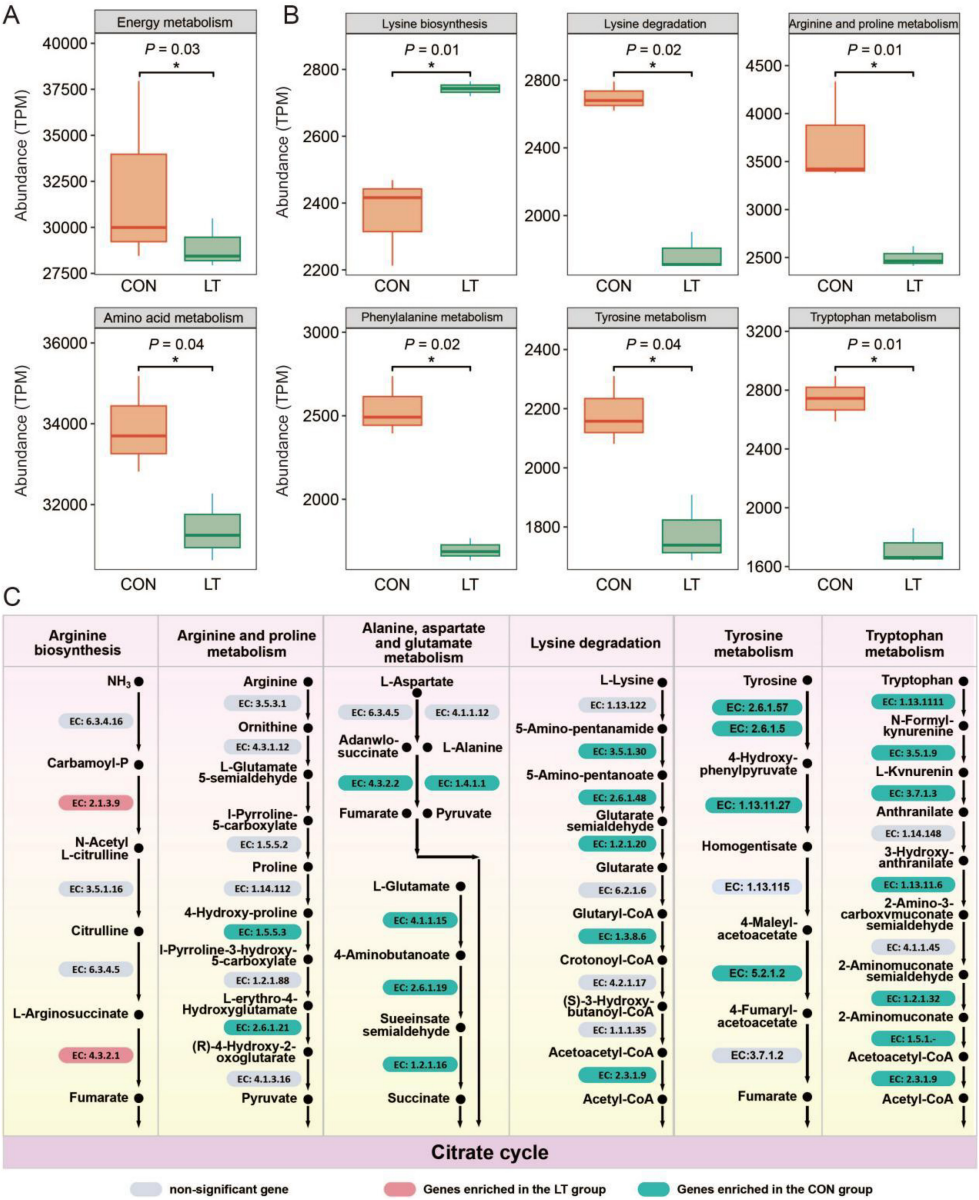
Comparative analysis of microbial functions based on KEGG pathways. **(A)** Comparison of energy metabolism and amino acid metabolism functions. **(B)** Comparison of different categories of amino acid metabolism functions. **(C)** Comparison of the predicted abundance of enzymes involved in different categories of amino acid metabolism. “*” indicates *P* < 0.05. CON, normal temperature group; LT, low-temperature group.

To further explore the impact of lower temperature on microbial energy and amino acid metabolism, we analyzed energy metabolism and amino acid metabolism of the holobiont using metagenomics. Notably, lower temperature significantly limited the energy and amino acid metabolic capacity of *E. sinensis* larval holobiont (*P* < 0.05, Figure 6A). More detailed analysis revealed that only lysine biosynthesis was elevated under lower temperature, whereas lysine degradation, arginine and proline metabolism, phenylalanine metabolism, tyrosine metabolism, and tryptophan metabolism were significantly suppressed (*P* < 0.05, Figure 6B). At the enzyme level, only EC:2.1.3.9 and EC:4.3.2.1 were upregulated under reduced temperature, primarily associated with arginine biosynthesis. Conversely, a set of enzymes were significantly downregulated under lower temperature, including EC:1.5.5.3 (arginine and proline metabolism); EC:4.3.2.2, EC:1.4.1.1, EC:4.1.1.15, EC:2.6.1.19, EC:1.2.1.16 (alanine, aspartate, and glutamate metabolism); EC:3.5.1.3, EC:2.6.1.48, EC:1.2.1.20, EC:1.3.8.6, EC:2.3.1.9 (lysine degradation); EC:2.6.1.57, EC:2.6.1.5, EC:1.13.11.27, EC:5.2.1.2 (tyrosine metabolism); and EC:1.13.11.1, EC:3.5.1.9, EC:3.7.1.3, EC:1.13.11.6, EC:1.2.1.32, EC:1.5. 1-, EC:2.3.1.9 (tryptophan metabolism). Notably, alterations in these amino acid metabolic pathways were closely associated with the citrate cycle, suggesting that reduced temperature markedly suppresses amino acid metabolism, consequently affecting energy metabolism (Figure 6C).

## Discussion

This study assessed the growth and developmental responses of *E. sinensis* larvae under temperature-driven conditions. Reduced temperature constrained larval development, a phenomenon commonly observed in crustaceans, primarily due to temperature-mediated effects on host metabolic rate and energy allocation. These findings underscore temperature as a key environmental determinant of developmental transitions (Lemos et al., 2003). Under lower temperature, larval overall length, head length, dorsal spine length, and frontal spine length were shorter, which may be closely related to the mechanism by which low temperature suppresses metabolic activity and limits energy allocation to growth tissues. It may be related to the developmental accumulated temperature required for larval growth and development (Ji et al., 2025; Keen et al., 2025). Furthermore, temperature may modulate crustacean larval survival and developmental success by shifting the prioritization of energy and resource allocation (Filgueira et al., 2016). Under reduced temperature, larvae are likely to expend more energy on maintaining basal metabolic processes, consequently reducing the proportion of energy available for growth (He et al., 2024; Salgado-García et al., 2023). This energy limitation not only delays developmental progression but may also lead to developmental abnormalities and compromise later survival, highlighting the pivotal regulatory role of temperature in *E. sinensis* larval development.

Host enzymatic activity serves as a crucial physiological indicator of metabolic homeostasis and energy utilization, playing a central regulatory role in larval growth and development (Zhang et al., 2023). AKP is generally associated with substance transport, phosphate metabolism, and non-specific immune defense, and its increased activity is commonly interpreted as an adaptive response to environmental stress (Wolinski et al., 2016; Zhang et al., 2021). ALT and AST, as key enzymes in amino acid and energy metabolism, and their decreased activity usually reflect a decline in overall metabolic rate and hepatopancreatic function, indicating that low temperature may suppress basal metabolic levels in larvae (Rojas et al., 2021). The decrease in TP indicates that protein synthesis capacity or amino acid utilization efficiency is inhibited, while GLU and TG, as important indicators of energy metabolism, are significantly reduced, reflecting that larvae face energy insufficiency or constrained energy mobilization under low temperature (Liu et al., 2019; Madeira et al., 2018). These changes in enzyme activities indicate that lower temperature may limit amino acid metabolism and energy metabolism in *E. sinensis* larvae.

The metabolic differences observed between the two temperature regimes suggest that reduced temperature may drive larvae into a distinct metabolic adaptive state, rather than simply suppressing overall metabolic activity (Lo et al., 2024). Furthermore, under reduced temperature, fewer metabolites were enriched, exhibiting a relatively concentrated profile, predominantly comprising molecules linked to stress responses (Caferro et al., 2025) and regulation of energy metabolism (Wu et al., 2026). By contrast, larvae maintained at normal temperature exhibited significant enrichment of diverse amino acids and their derivatives, reflecting a more active and heterogeneous metabolic profile, in line with the observed acceleration of growth and development. Under these conditions, differential metabolites were predominantly enriched in pathways such as lysine biosynthesis and degradation, arginine biosynthesis, phenylalanine, tyrosine, and tryptophan metabolism, as well as alanine, aspartate, and glutamate metabolism. These pathways are pivotal for protein synthesis, energy provision, and signaling molecule production, processes closely linked to accelerated growth and tissue differentiation (Wu et al., 2021; Zhao et al., 2022). By contrast, under reduced temperature, differential metabolites were predominantly enriched in arginine and proline metabolism, a pathway linked to stress adaptation, osmotic regulation, and oxidative stress defense, indicating that larvae may prioritize survival-oriented metabolic strategies over growth-promoting processes in response to low temperature (Feng et al., 2020; Huo et al., 2023; Liu et al., 2025).

These findings indicate that temperature shifts do not merely suppress metabolic activity in *E. sinensis* larvae; instead, they reprogram amino acid–centered metabolic networks, thereby modulating energy allocation and the prioritization of physiological processes (García et al., 2023; Li and Zhang, 2026). Moreover, the increased AVD observed in the low-temperature group suggests heightened internal heterogeneity within the holobiont and a shift toward ecological instability under reduced temperature (Kong et al., 2023; Zhang et al., 2025). Under reduced temperature, the Beta deviation index increased and the habitat niche breadth decreased, suggesting that lower temperature may strengthen environmental filtering within the microbial community, thereby narrowing ecological niches and diminishing functional redundancy (McAteer et al., 2020). Meanwhile, under low-temperature conditions, the proportion of persistent microorganisms increased while intermittent taxa decreased, suggesting that the holobiont may gradually become dominated by a few stable, stress-tolerant taxa. This community structure pattern may be closely associated with a reduction in metabolic flexibility (Mu et al., 2024; Wang et al., 2020).

In this study, reduced temperatures limited the growth of *Actinomycetota* (Bao et al., 2021), *Bacteroidota* (Lindh et al., 2015), and *Thermodesulfobacteriota* (Luo et al., 2025)—taxa typically involved in organic matter degradation, amino acid conversion, and nitrogen cycling—potentially impairing the holobiont’s capacity to contribute to host nutrient metabolism. Under normal temperature conditions, *Roseovarius*, *Demequina*, *Methylobacterium*, *Acinetobacter*, and *Ruegeria* were enriched, which are mostly associated with organic matter oxidation, nitrogen transformation, and amino acid metabolism (Humaira et al., 2024; Stirrup et al., 2023; Zecher et al., 2020; Zhang S. et al., 2024; Zhou J. et al., 2022), whereas *Leucothrix*, *Marivita*, *Yoonia*, and *Pseudoalteromonas* were enriched under low-temperature conditions and tended to be environment-adaptive or stress-tolerant taxa (Baek et al., 2018; Wang et al., 2023; Xu et al., 2021; Zheng et al., 2021). Notably, 99 specific ASVs were present under both temperature conditions, suggesting that temperature variation not only reshapes the dominant taxa but may also drive the reassembly of the holobiont community.

In this study, low temperature significantly inhibited fermentation and multiple nitrogen transformation–related functions, including nitrate respiration, nitrite respiration, and denitrification, whereas hydrocarbon degradation was enhanced, further indicating that the holobiont under lower temperatures may shift from efficient energy and nitrogen cycling to a low-energy maintenance–oriented metabolism, which may directly affect the host’s efficiency in acquiring nitrogen sources and energy substrates (Gao et al., 2024; Wen et al., 2020; Zhang C. et al., 2024). Metagenomic analyses further demonstrated that reduced temperature significantly diminished the overall energy metabolism and amino acid metabolic capacity of the holobiont. Notably, multiple rate-limiting enzymes across amino acid metabolic pathways-including lysine degradation, arginine and proline metabolism, phenylalanine metabolism, tyrosine metabolism, and tryptophan metabolism-were markedly downregulated under low-temperature conditions; these pathways are tightly coupled to the TCA cycle (Hong et al., 2023; Li et al., 2022; Ryan et al., 2021; Suzuki et al., 2021). This suggests that low temperature may weaken the ability of the microbiota to supply energy substrates to the host by inhibiting microbial amino acid degradation and transformation processes, thereby affecting host energy metabolism and growth and development (Hu et al., 2025; Niu et al., 2025; Zhao et al., 2024).

Although this study revealed the association between amino acid metabolism and microbial functional remodeling in larvae under low temperature through multi-omics analyses, several limitations should be acknowledged. First, variation in larval developmental stages, which was difficult to fully standardize, may have substantially influenced host metabolism and microbial community composition. In addition, due to the small body size and frequent movement of larvae, it was challenging to continuously and accurately monitor larval survival across replicates, resulting in the absence of survival rate data and limiting a comprehensive assessment of the ecological consequences of low-temperature adaptation. Future studies should incorporate multiple temperature gradients and apply more refined techniques to monitor larval survival and metabolic changes, which will help further elucidate the response mechanisms of crustacean larvae to environmental temperature fluctuations.

## Conclusion

This study provides new insights into how water temperature shapes growth and metabolic programming in ectothermic crustacean larvae. Reduced temperature constrained life-history progression in *E. sinensis* larvae, limiting both overall metabolic activity and energy utilization. Temperature fluctuations remodeled metabolic flux in *E. sinensis* larvae, reshaping holobiont community structure and ecological strategies, attenuating nitrogen-cycling functions, and suppressing microbial amino acid metabolism potential. These changes collectively impaired the TCA cycle, restricted energy production, and ultimately constrained larval growth and development. Technical constraints prevented the quantification of larval survival rates, a key fitness trait for aquaculture applications, To address these gaps, future advancements should focus on this aspect. Future studies should employ fine-scale temperature gradients and integrative approaches, such as transcriptomics, to elucidate the mechanisms by which crustacean larvae respond to water temperature fluctuations, thereby advancing our understanding of larval-environment interactions.

## Data Availability

The original contributions presented in this study are included in the article/supplementary material, further inquiries can be directed to the corresponding author.

## Footnotes

1
https://cloud.majorbio.com/

2
http://www.cazy.org/
